# Datasets of microarray analysis to identify *Gpr137b*-dependent interleukin-4-responsive genes in the mouse macrophage cell line RAW264

**DOI:** 10.1016/j.dib.2019.01.017

**Published:** 2019-01-21

**Authors:** Zohirul Islam, Aya Horikawa, Takashi Inui, Osamu Ishibashi

**Affiliations:** Laboratory of Biological Macromolecules, Graduate School of Life and Environmental Sciences, Osaka Prefecture University, Japan

## Abstract

Macrophages are classified mainly into two subtypes, M1 and M2, which exhibit distinct phenotypes, based on their microenvironment. We have recently demonstrated that *Gpr137b* is abundantly expressed in RAW264 macrophages, “*Gpr137b* is an orphan G-protein-coupled receptor associated with M2 macrophage polarization” (Islam et al., in press) [1]. Although recent studies have suggested that G-protein-coupled receptors (GPCRs) are associated with M1/M2 macrophage polarization (“G-protein-coupled bile acid receptor 1 (GPBAR1, TGR5) agonists reduce the production of proinflammatory cytokines and stabilize the alternative macrophage phenotype” (Hogenauer et al., 2014) [2], “Leukotriene B4 promotes neovascularization and macrophage recruitment in murine wet-type AMD models” (Sasaki et al., 2018) [3]), available information about GPCR-mediated macrophage polarization is still limited. This prompted us to generate *Gpr137b*-knockout (KO) RAW264 clones using the CRISPR/Cas9 genome editing system to elucidate the function of *Gpr137b* in interleukin (IL)-4-induced M2 macrophage polarization (Islam et al., in press) [1].

Here we present the datasets of a microarray analysis to identify *Gpr137b*-dependent IL-4-responsive genes in RAW264 cells. The raw microarray data are available in the Gene Expression Omnibus database (https://www.ncbi.nlm.nih.gov/geo/) under the accession number GSE117578, https://www.ncbi.nlm.nih.gov/geo/query/acc.cgi?acc=GSE117578.

**Specifications table**

**Table:** 

Subject area	Biology
More specific subject area	Transcriptomics
Type of data	Figures, Microarray data
How data was acquired	Affymetrix Clariom S Assay, mouse
Data format	Raw data (CEL files)
Experimental factors	*Gpr137b*-knockout and wildtype RAW264 macrophages with or without interleukin-4 treatment
Experimental features	Two *Gpr137b*-knockout and two wildtype clones of RAW264 macrophages were established using the CRISPR/Cas9 genome editing system. These clones were treated with or without interleukin-4 and subjected to microarray-based gene expression analysis.
Data source location	Osaka Prefecture University, Sakai, Japan
Data accessibility	The microarray datasets in this article are available through Gene Expression Omnibus database (NCBI) (Accession Number GSE117578).
Related research article	Zohirul Islam, Takashi Inui and Osamu Ishibashi. *Gpr137b* is an orphan G-protein-coupled receptor associated with M2 macrophage polarization. Biochemical and Biophysical Research Communications, in press [1].

**Value of the data**

• Signaling pathways involving *Gpr137b* in IL-4-induced M2 macrophage polarization can be clarified.
• The datasets enrich information about M2 macrophage polarization-associated genes.
• The datasets can provide novel insights into the macrophage-related post-inflammation tissue repair.

## 1 Data

**Fig. 1:**
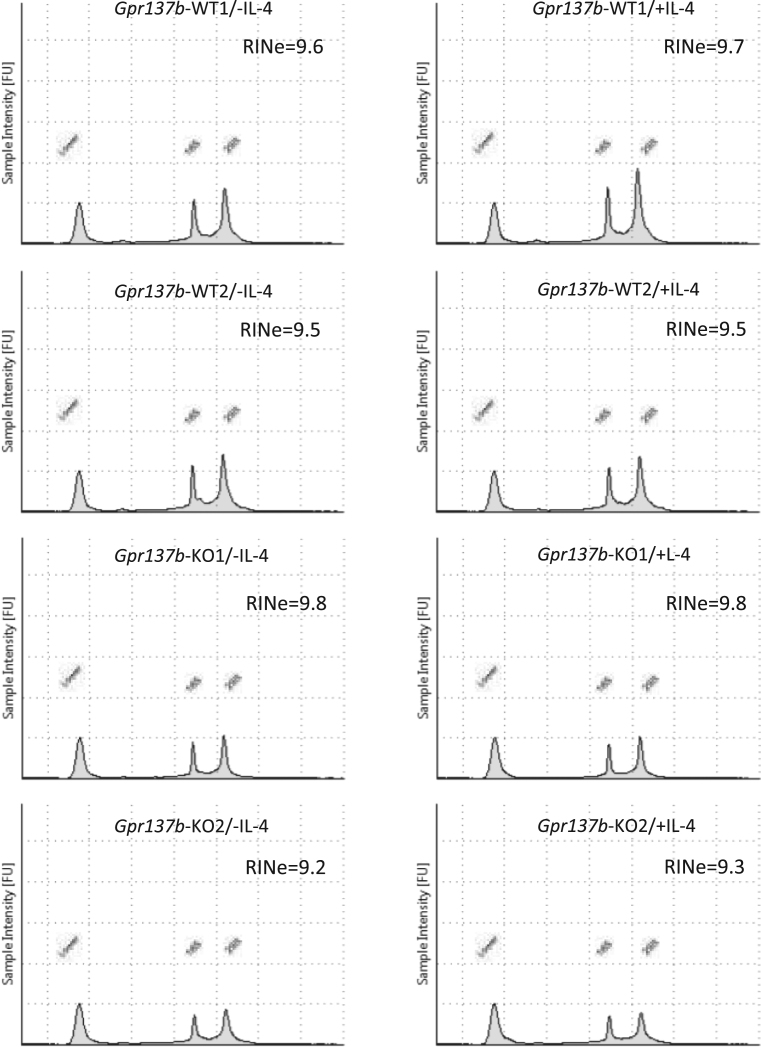
RNA integrity number equivalent (RINe) values of RNA samples used for microarray analysis.

RNA quality was assessed by RNA integrity number equivalent (RINe), a representative index to assess RNA quality. The RINe values of all RNA samples used for the experiment were more than 9.2 (Fig. 1). To visualize differential gene expression among the experimental groups a heatmap was generated from normalized data using the Affymetrix® Transcriptome Analysis Console 4.0 software (Supplementary file 1). Microarray and sample annotation data were deposited in Gene Expression Omnibus under accession number GSE117578. Direct link to the deposited data is available at https://www.ncbi.nlm.nih.gov/geo/query/acc.cgi?acc=GSE117578.

## 2 Experimental design, materials and methods

### 2.1 Cell culture

RAW264 macrophages were obtained from RIKEN Bioresource Center (Tsukuba, Japan) and grown in EMEM culture medium (Wako, Osaka, Japan) supplemented with 10% fetal bovine serum (Thermo-Fischer, Waltham, MA, USA) and Antibiotic-Antimycotic (Sigma-Aldrich, St. Louise, MO, USA); it was then incubated in a humidified atmosphere with 5% CO_2_ at 37 °C. When the cells were treated with IL-4, the culture medium was replaced with serum-deprived and 0.1% BSA-containing EMEM medium to reduce background.

### 2.2 RNA isolation

Total RNA was isolated using RNAiso-Plus reagent (Takara-bio, Kusatsu, Japan) according to the manufacturer׳s protocol. RINe values were determined using Agilent 2200 TapeStation (Agilent, Santa Clara, CA, USA).

### 2.3 Microarray analysis

Comprehensive gene expression analysis was performed using the Clariom S Assay, mouse (Thermo-Fischer). Gpr137b-wildtype (WT) and -KO RAW264 cells (2 independent clones for each) with or without IL-4 treatment for 48 h were subjected to this analysis. A hundred ng of RNA was used for the following labeling reaction. cRNA and single strand (ss) cDNA were synthesized using Affymetrix® GeneChip® WT Plus Reagent according to the manufacturer׳s instruction. The ssDNA (5.5 μg) was then fragmented and biotin-labeled using GeneChip^®^ WT Terminal Labeling Kit (Thermo-Fischer) according to the manufacturer׳s manual. Labeled cRNA was hybridized for 17 h on the microarray using GeneChip Hybridization, Wash, and Stain Kit (Thermo-Fischer). To visualize fluorescence signals the microarray was scanned using the GeneChip^®^ Scanner 3000 7G.

### 2.4 Data processing

**Fig. 2:**
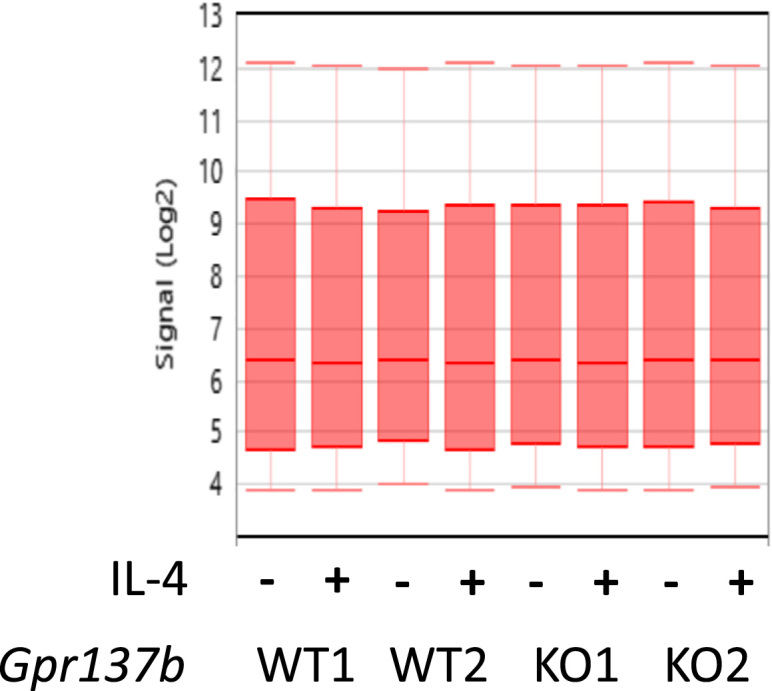
Signal box plots of array files after CHP normalization.

The quality of the experiment was assessed based on the values of pos vs neg auc and pm mean, which were calculated using the Affymetrix^®^ Expression Console software (Thermo-Fischer) (Table 1). CEL files were processed for each replicate and experimental condition using the Affymetrix^®^ Transcriptome Analysis Console software 4.0 (Thermo-Fischer). The CEL files were then subjected to normalization using the Signal Space Transformation-Robust Multiarray Analysis (SST-RMA) method [4] to generate CHP files. The comparability of the relative log expression signal across all samples are ensured (Fig. 2).

**Table 1:** Microarray outliers.

**Sample**	**GEO sample accession**	**pm_mean**	**pos_vs_neg_auc**
Gpr137b-WT1/-IL-4	GSM3304078	333.9	0.8257
Gpr137b-WT1/+IL-4	GSM3304079	474.5	0.8215
Gpr137b-WT2/-IL-4	GSM3304080	437.0	0.8050
Gpr137b-WT2/+IL-4	GSM3304081	367.4	0.8299
Gpr137b-KO1/-IL-4	GSM3304082	366.3	0.8150
Gpr137b-KO1/+IL-4	GSM3304083	366.8	0.8256
Gpr137b-KO2/-IL-4	GSM3304084	369.7	0.8202
Gpr137b-KO2/+IL-4	GSM3304085	377.5	0.8184

PM_mean, a probe-level metric, is the mean of perfect match raw intensities prior to data normalization. Pos_vs_neg_auc is the area under the curve (AUC) for a receiver operating characteristic (ROC) plot comparing signal values for the positive controls to the negative controls, and is a robust measure of the global quality of the data.

## Figures and Tables

**Fig. S1:**
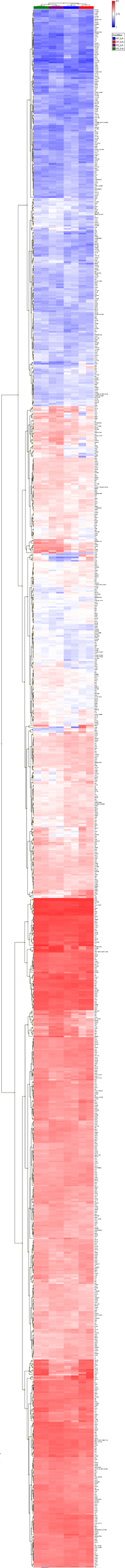
Heatmap visualizing gene expression in *Gpr137b*-WT and -KO RAW264 cells treated with or without IL-4. Hierarchical clustering of the genes and samples is also shown.
